# Posterior tibial slope and static anterior tibial translation are not associated with increased cyclops syndrome after anterior cruciate ligament reconstruction

**DOI:** 10.1002/jeo2.70664

**Published:** 2026-04-23

**Authors:** David Mazy, Nicolas Cance, Lucia Angelelli, Tomas Pineda, Andrea Pintore, David Henri Dejour

**Affiliations:** ^1^ Lyon Ortho Clinic, Orthopedic Surgery Department Clinique de la Sauvegarde Lyon France; ^2^ Clinica Ortopedica e Traumatologica 2 IRCCS Istituto Ortopedico Rizzoli Bologna Italy; ^3^ Facultad de Medicina, Universidad Andrés Bello Hospital del Trabajador Santiago Chile; ^4^ Facultad de Medicina, Universidad Finis Terrae Hospital el Carmen Santiago Chile; ^5^ Department of Trauma and Orthopaedic Surgery Istituto Clinico Mediterraneo ICM Agropoli SA Italy

**Keywords:** anterior cruciate ligament reconstruction, cyclops, cyclops syndrome, posterior tibial slope, static anterior tibial translation

## Abstract

**Purpose:**

Posterior tibial slope (PTS) and static anterior tibial translation (SATT) are established risk factors for anterior cruciate ligament (ACL) graft rupture and may also be associated with cyclops syndrome. This study aimed to assess whether these anatomical parameters influence the prevalence of cyclops syndrome after ACL reconstruction (ACLR). The hypothesis of the present study was that increased PTS and SATT would facilitate the development of cyclops syndrome.

**Methods:**

Patients aged ≥14 years with a minimum follow‐up of 6 years who underwent primary ACLR with hamstring autograft between January 2015 and December 2017 were included. Demographic data, PTS, SATT, concomitant lateral extra‐articular tenodesis (LET) and reoperation for cyclops syndrome were recorded. Time from index surgery to arthrolysis was documented, with a minimum follow‐up of 6 years. Subgroup analysis regarding PTS, SATT and gender was performed. Univariate and multivariate logistic regression analyses were conducted to identify independent risk factors.

**Results:**

Of 530 patients included for analysis, 18 (3.4%) developed cyclops syndrome at a mean of 14 ± 9 months postoperatively (range, 6–33 months). Patients with a PTS ≥ 12° had a 4.3% rate of cyclops syndrome compared with 3.2% in those with a PTS < 12° (*p* = 0.536). Patients with a SATT ≥ 5 mm had a 5.6% rate compared with 2.7% for SATT < 5 mm (*p* = 0.154). Female patients presented a statistically significant higher prevalence (5.9%) compared with males (1.8%, *p* = 0.024). Concomitant LET did not demonstrate a protective effect (*p* = 0.807). Female sex was the only independent predictor of cyclops syndrome (OR, 3.3; 95% CI, 1.2–9.1; *p* = 0.018).

**Conclusion:**

This study found no evidence that increased PTS or SATT predisposes to cyclops syndrome after ACLR with hamstring autograft. These preoperative parameters should not alert clinicians to an increased risk of postoperative cyclops syndrome.

**Level of Evidence:**

Level III, retrospective case‐control study.

## Graphical abstract

This study found no evidence that increased posterior tibial slope or static anterior tibial translation predisposes to cyclops syndrome after ACLR with hamstring autograft.

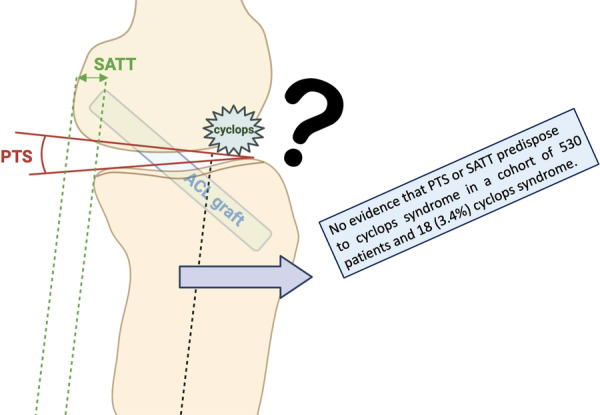

**Abbreviations**
ACL: anterior cruciate ligament
ACLR: anterior cruciate ligament reconstruction
DATT: dynamic anterior tibial translation
LEAP: lateral extra‐articular procedure
LET: lateral extra‐articular tenodesis
PTS: posterior tibial slope
SATT: static anterior tibial translation

## INTRODUCTION

Anterior cruciate ligament (ACL) rupture is a frequent sports‐related injury, most often requiring ACL reconstruction (ACLR) [4]. Among postoperative complications, cyclops syndrome is a well‐recognised cause of extension deficit and/or pain at terminal extension, sometimes associated with an audible or palpable clunk due to a fibrous nodule within the intercondylar notch or at the tibial tunnel [16, 36]. Other complications after ACLR include graft rupture, stiffness, osteoarthritis, persistent laxity, and, more rarely, infection, neurovascular injury or thromboembolic events [17, 25]. Cyclops syndrome has been reported in 2%–10% of cases following ACLR [21]. Cyclops syndrome must be distinguished from a cyclops lesion, which is defined radiologically as a nodular mass in the intercondylar notch on magnetic resonance imaging (MRI), the current gold standard for diagnosis [21]. A cyclops lesion is not necessarily symptomatic, whereas cyclops syndrome refers specifically to a symptomatic lesion [36]. The prevalence of cyclops lesions ranges from 15% to 35% arthroscopically and from 33% to 46.8% on MRI [1, 13, 29]. Several risk factors for cyclops syndrome have been reported, including early postoperative extension deficit, bone‐patellar tendon‐bone graft, notch morphology and patient‐related factors such as older age and sex [19, 20, 36]. Female sex has been associated with a higher risk of arthrofibrosis and cyclops syndrome in some series, potentially related to morphological differences such as narrower intercondylar notches [21, 36]. Cyclops syndrome can hinder rehabilitation, reduce functional outcomes and generally requires arthrolysis with resection of the lesion [21, 33]. However, when revision surgery is necessary, postoperative range of motion (ROM) recovery is often disappointing [26]. Better outcomes are achieved if arthrolysis is performed within 1 year of ACLR and followed by structured rehabilitation [10]. Posterior tibial slope (PTS) and static anterior tibial translation (SATT) are recognised risk factors for ACL graft rupture, as they represent anterior‐posterior translation and the mechanical load applied on the graft [3, 18]. Repetitive microtrauma to the graft has been suggested as a potential mechanism contributing to cyclops syndrome formation [21]. In this context, increased PTS may further accentuate anterior tibial translation and potentially promote repetitive microtrauma, and one recent study has suggested an association between increased PTS and cyclops syndrome [5]. However, the role of SATT in the development of cyclops syndrome remains unclear.

The purpose of this study was to investigate the relationship between PTS, SATT and the incidence of cyclops syndrome after ACLR. The hypothesis of the present study was that increased PTS and SATT would facilitate the development of cyclops syndrome.

## METHODS

### Ethics

All patients provided informed consent for the use of their data for research purposes, and the study was approved by the institutional ethical board (No. COS‐RGDS‐2020‐03‐006‐DEJOUR‐D).

### Study design

A retrospective analysis was conducted on a consecutive series of ACLR performed in a sports knee referral centre between January 2015 and December 2017. Inclusion criteria were primary single‐bundle ACLR using hamstring tendon autograft, age ≥ 14 years, and a minimum follow‐up of 6 years. Exclusion criteria included additional associated procedures (such as slope‐reducing or coronal plane osteotomies, cartilage surgery), revision ACLR, multiligament knee injuries, chondral injuries requiring surgical treatment or arthrolysis for stiffness without evidence of a cyclops lesion. At the last follow‐up, if the patient reported having undergone surgery for ACL graft rupture without a previous cyclops syndrome diagnosis, the patient was excluded from this study. An ACL graft rupture may mask an underlying cyclops lesion that will not have the opportunity to be diagnosed.

### Surgical technique

All patients underwent ACLR with pedicular hamstring tendon autograft. The semitendinosus and gracilis tendons were harvested using an open stripper. Femoral and tibial fixation were achieved with bioabsorbable interference screws (Ligafix; SBM). The femoral tunnel was positioned centrally to reproduce both bundles through a single‐bundle reconstruction, performed using an outside‐in technique. A lateral extra‐articular tenodesis (LET) was systematically performed in patients younger than 18 years of age. In patients older than 18 years, LET was selectively performed in the presence of genu recurvatum >10°, hyperlaxity or pivot‐shift grade 2 or 3 [15]. LET was performed using the modified Lemaire technique, in which a 1 × 9 cm strip of the posterior iliotibial band was harvested, passed deep to the lateral collateral ligament, and fixed with a 7 mm interference screw (Ligafix; SBM) in a femoral tunnel positioned 5 mm proximal and 5 mm posterior to the lateral collateral ligament insertion, with the knee at 80° of flexion and in neutral rotation [8].

### Postoperative management

All patients were discharged home on the day of surgery. A standardised rehabilitation program was followed, beginning on postoperative day one with isometric quadriceps activation and passive and active ROM exercises from 0° to 90°, with a primary emphasis on restoring full knee extension. ROM was progressively advanced to achieve full ROM by 6 weeks. No brace was used. Weight‐bearing as tolerated with crutches was permitted. Patients undergoing meniscal repair for radial or root tears followed a non‐weight‐bearing protocol, with flexion limited to 90° during the first 3 weeks. Return to sports was generally permitted at 9 months, based on functional and isokinetic testing.

### Cyclops syndrome definition

**Figure 1:**
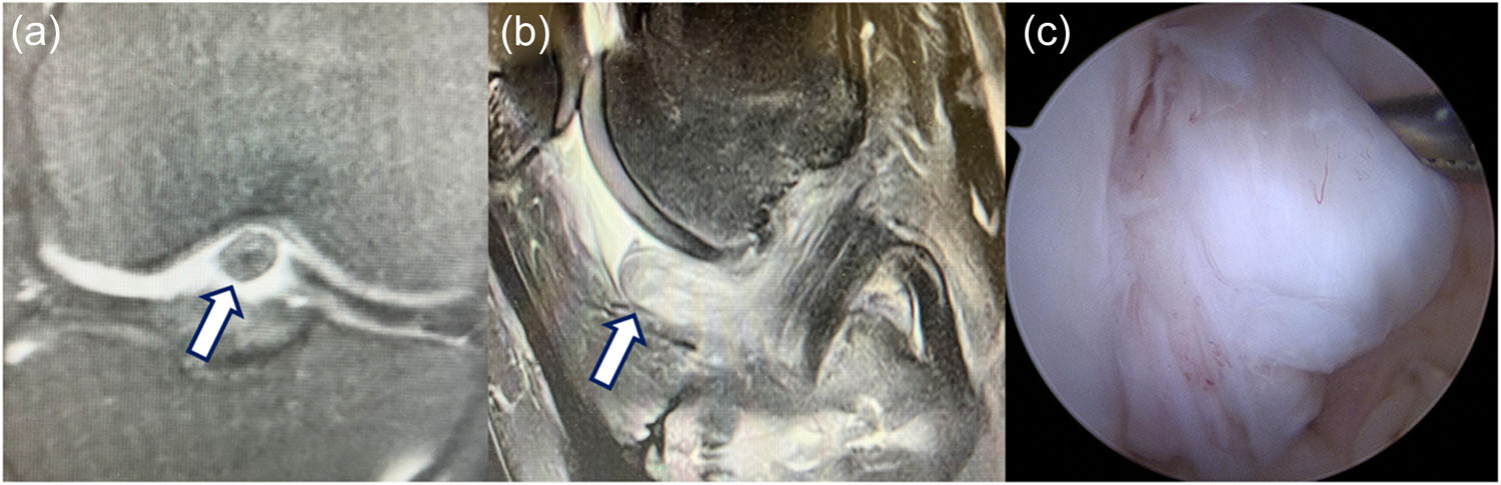
Cyclops syndrome identified 9 months after anterior cruciate ligament reconstruction. (a) Frontal plane magnetic resonance imaging (MRI). (b) Sagittal plane MRI. (c) Arthroscopic view of the fibrous nodule within the intercondylar notch. Arrows indicate the cyclops lesion.

Cyclops syndrome was defined as a postoperative extension deficit and/or pain at terminal extension after ACLR, in association with MRI evidence of a fibrous nodule located either in the intercondylar notch or at the tibial tunnel, requiring arthroscopic arthrolysis (Figure 1). No minimum size threshold for the lesion was applied. At arthroscopy, the fibrous nodule had to be clearly identified and mechanical impingement during full knee extension had to be observed.

Identification of asymptomatic cyclops lesions would require systematic postoperative MRI, which is not routinely performed in our institution. Therefore, the present study deliberately focused on clinically symptomatic cyclops syndrome.

### Data collection

All data were prospectively entered into our institutional registry. Recorded variables included patient age at the time of the ACLR, sex, laterality, last follow‐up, meniscus surgeries (repair or meniscectomy) and concomitant LET. Preoperative knee extension deficit was recorded in patients who developed cyclops syndrome. In cases of cyclops syndrome, the time interval between ACLR and diagnosis was also documented. Over the follow‐up period, any recurrence of cyclops syndrome was also specifically investigated.

### Imaging assessment

All patients underwent standardised radiographic evaluation at the same institution and radiology department. Radiographs consisted of an anteroposterior view and a true lateral view of the knee in monopodal weight‐bearing at 20° of flexion, with a minimum of 15 cm of proximal tibia visible. Measurements of PTS and SATT were performed on preoperative radiographs using HOROs DICOM viewer software (version 3.3.6). The PTS was assessed using the proximal anatomical axis method, defined as the angle between the perpendicular to the tibial diaphysis and the tangent to the anterior and posterior margins of the medial tibial plateau [2]. SATT was defined as the distance between two lines parallel to the posterior tibial cortex: the first tangent to the posterior border of the medial tibial plateau, and the second tangent to the posterior border of the femoral condyles [3].

### Patient follow‐up

At a minimum follow‐up of 6 years, patients were contacted by phone and email to identify any arthrolysis for cyclops syndrome following their primary ACLR. Patients were considered lost to follow‐up if no response was obtained within a 6‐month period. The follow‐up duration reported corresponds to the longest available in our database. It is noteworthy that most procedures for arthrofibrosis and cyclops syndrome occur within the first two postoperative years [28, 29].

### Subgroup analysis

Patients were stratified according to a PTS threshold of 12° and an SATT threshold of 5 mm [7, 18]. These values have previously been identified as clinically relevant thresholds associated with an increased risk of ACLR graft rupture [23, 27, 34]. These thresholds were intentionally adopted to maintain consistency with existing literature and avoid introducing arbitrary new values. Additional comparisons were performed for the entire cohort based on gender.

### Statistical analysis

Continuous variables with normal distribution are presented as mean ± standard deviation, while categorical variables are reported as absolute numbers and percentages. Normality of continuous data was assessed using the Shapiro–Wilk test. For group comparisons, the chi‐square test or Fisher's exact test (when frequencies were <5%) was used for categorical variables, and either independent‐samples *t*‐test or Mann–Whitney *U‐*test was used for continuous variables, depending on the normality assessment. Risk factors for cyclops syndrome were first analysed by univariate testing, with variables showing *p* < 0.20 subsequently included in a multivariable logistic regression model. Odds ratios (OR) with 95% confidence intervals (CI) were reported. Fifty radiographs were independently reviewed to calculate the intraclass correlation coefficient (ICC) for PTS and SATT by two examiners (D.M. and L.A.). Intraobserver reliability was evaluated by repeating measurements twice by one examiner (D.M.) with a 2‐week interval. Statistical analyses were performed using SPSS Statistics (version 29.0.1.0; IBM Corp.), and statistical significance was set at *p* < 0.05.

## RESULTS

### Population

**Figure 2:**
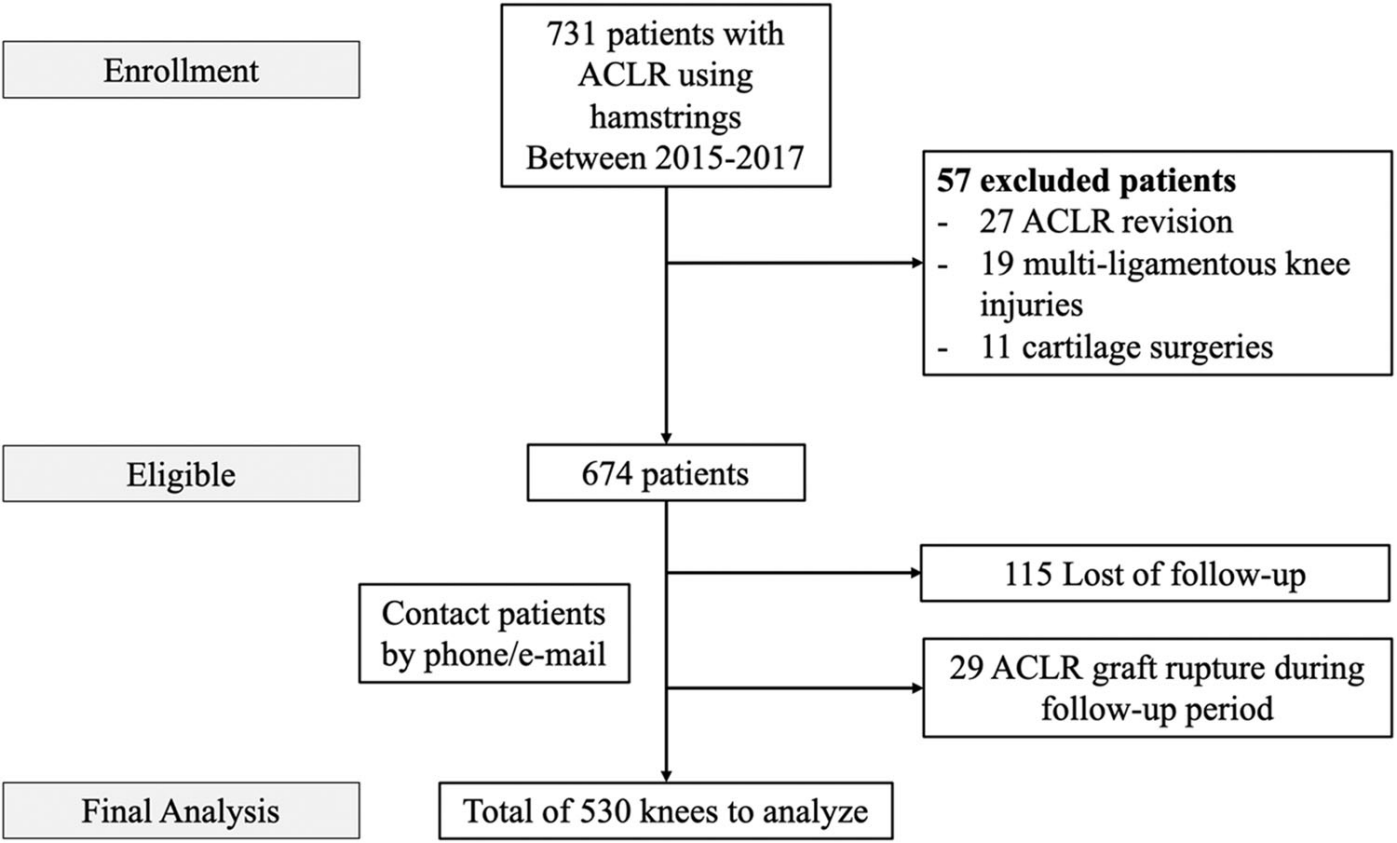
Study flowchart. ACLR, anterior cruciate ligament reconstruction.

A total of 674 patients met the inclusion criteria. Of these, 115 were lost to follow‐up and 29 experienced graft rupture during the follow‐up period, leaving 530 patients available for analysis. The study flowchart is presented in Figure 2. Eighteen patients (3.4%) developed cyclops syndrome during the study period. No recurrence of cyclops syndrome was observed during the follow‐up period. Sixteen of the 18 patients who developed cyclops syndrome presented with an extension deficit, with a mean deficit of 8.3° ± 3.5°. The remaining two patients with no extension deficit reported painful terminal extension limiting sports activities, with cyclops lesions confirmed on MRI. The mean follow‐up was 94 ± 16 months (range, 72–108 months), and the mean time to cyclops syndrome was 14 ± 9 months postoperatively (range, 6–33 months). No cyclops syndrome was observed beyond 3 years after ACLR. The patient who underwent arthrolysis at 33 months postoperatively initially reported only mild extension‐end pain without extension deficit and declined reoperation. He later returned when attempting to go back to high‐level sports activity and underwent surgery. Demographic characteristics and radiographic measurements of the cohort are presented in Table 1.

**Table 1:** Patients' demographics and radiographic measurements.

Demographics	Mean (*n* = 530)	SD	[Min–max]
Age (years)	31	11	[14–71]
Gender (female)	204 (38%)		
Side (right)	283 (53%)		
LET	161 (30%)		
Cyclops syndrome	18 (3.4%)		
Partial meniscectomy MM/LM	65/57		
Meniscal Repair MM/ML	154/88		
Radiographic measurements			
PTS (°)	9.2	2.5	[2–18]
SATT (mm)	2.3	3.4	[−8 to 14]

Abbreviations: LET, lateral extra‐articular tenodesis; LM, lateral meniscus; MM, medial meniscus; PTS, posterior tibial slope; SATT, static anterior tibial translation.

### Subgroups analysis

The results of group comparisons according to the PTS and SATT thresholds are presented in Tables 2 and 3.

**Table 2:** Comparison of patients using a PTS threshold of 12°.

	PTS < 12° (*n* = 437)	PTS ≥ 12°(*n* = 93)	*p*‐value
Age (years)	31 ± 11	30 ± 11	0.321
Gender (female %)	166 (38%)	38 (41%)	0.605
Side (right %)	235 (54%)	48 (52%)	0.704
Cyclops syndrome	14 (3.2%)	4 (4.3%)	0.536

Abbreviation: PTS, posterior tibial slope.

**Table 3:** Comparison of patients using a SATT threshold of 5 mm.

	SATT < 5 mm (*n* = 405)	SATT ≥ 5 mm (*n* = 125)	*p*‐value
Age (years)	31 ± 11	30 ± 11	0.300
Gender (female %)	160 (40%)	44 (35%)	0.387
Side (right %)	218 (54%)	65 (52%)	0.720
Cyclops syndrome	11 (2.7%)	7 (5.6%)	0.154

Abbreviation: SATT, static anterior tibial translation.

### Gender comparison

Female patients demonstrated a higher prevalence of cyclops syndrome compared with male patients, whereas no significant differences were observed for age, PTS or SATT (Table 4).

**Table 4:** Gender comparison.

	Female (*n* = 204)	Male (*n* = 326)	*p*‐value
Age (years)	31 ± 13	31 ± 9	0.530
PTS	9.3 ± 2.4	9.1 ± 2.5	0.376
SATT	2.2 ± 3.1	2.4 ± 3.6	0.607
Cyclops syndrome	12 (5.9%)	6 (1.8%)	**0.024**

*Note*: Bold value indicates statistical significance at *p* < 0.05.

Abbreviations: PTS, posterior tibial slope; SATT, static anterior tibial translation.

### Risk factors analysis

Univariate logistic regression analysis demonstrated that PTS ≥ 12° (*p* = 0.597), SATT ≥ 5 mm (*p* = 0.128), age < 18 years (*p* = 0.597) and the presence of a LET (*p* = 0.807) were not significantly associated with the occurrence of cyclops syndrome. Female sex was the only variable significantly associated with cyclops syndrome (OR, 3.3; 95% CI, 1.2–9.1; *p* = 0.018).

A post hoc power analysis was conducted at a significance level of *α* = 0.05 to evaluate whether the available sample size was sufficient to detect a difference in cyclops syndrome prevalence between genders. Based on the observed proportions, the analysis demonstrated a statistical power of 72.4%, indicating a moderate probability of detecting the observed association.

There were excellent intra and interobserver reliability ICCs for PTS (0.940 and 0.923, respectively) and SATT (0.950 and 0.915, respectively).

## DISCUSSION

The most important finding of this study is that neither PTS nor SATT influenced the risk of developing cyclops syndrome following ACLR with hamstring autograft. As these parameters are measured preoperatively, they should not prompt clinicians to anticipate a higher postoperative risk of cyclops syndrome. The incidence of cyclops syndrome in this study (3.4%) is consistent with previous reports, which estimate its prevalence between 2% and 10% after ACLR [20, 21, 36].

Cong et al. identified PTS as a risk factor for cyclops syndrome but did not evaluate SATT [5]. The discrepancies between their findings and ours may be explained by methodological differences, particularly in radiographic measurements and cohort characteristics, including notch morphology, graft type, tunnel positioning and the presence of knee hyperextension. These variations underscore the importance of comprehensive data collection, as the risk factors for cyclops syndrome remain highly controversial and are likely multifactorial. PTS and SATT, measured on preoperative lateral radiographs, are recognised risk factors for ACL graft failure, but they do not appear to predict the occurrence of cyclops syndrome [18, 27]. These parameters reflect the anterior guided force vector acting on the graft. Contrary to the initial hypothesis, increased PTS or SATT did not increase the risk of cyclops syndrome. One possible explanation is that SATT was measured preoperatively, whereas postoperative SATT is typically partially reduced following graft placement [3, 31]. Assessing cyclops prevalence in relation to postoperative SATT could therefore provide more relevant insights. Another possible explanation is that these factors may predispose patients to the formation of cyclops lesions detectable on MRI but not necessarily to the development of symptomatic cyclops syndrome. Future studies directly comparing radiological and clinical outcomes are required to clarify whether PTS or SATT influence the rate of cyclops lesions that remain asymptomatic. Assessment of cyclops syndrome should primarily remain clinically driven and subsequently supported by imaging, rather than based on sagittal radiographic parameters [21].

In this study, female sex was the only independent risk factor identified. Female sex has been reported as a risk factor for both arthrofibrosis and cyclops syndrome [6, 11, 22, 28]. The mechanisms underlying this association remain unclear. Hypotheses include differences in return to pre‐injury activity levels, variations in the intensity and timing of rehabilitation after ACLR. Alternatively, it has been suggested that women may be more likely to seek medical attention for residual extension deficits, thus increasing the likelihood of diagnosis and intervention [28]. From a morphological perspective, women generally present with narrower intercondylar notches, which may also contribute to the higher prevalence of cyclops syndrome [5, 24, 30].

In this study, LET did not confer protection against cyclops syndrome. Recent data suggest that lateral extra‐articular procedure (LEAP) procedures may reduce the risk of recurrent cyclops syndrome but not necessarily for primary cyclops [19]. Voskuijl et al. reported an increased prevalence of cyclops syndrome when a modified Ellison technique was used for lateral extra‐articular procedures [35]. Differences in surgical technique may therefore limit direct comparison with these results. LEAP improved anterolateral stability and reduced rotational stress on the graft [12]. This distinction between primary and recurrent cyclops syndrome warrants further investigation in larger comparative cohorts.

Graft type has been identified as another potential risk factor. Patellar and quadriceps tendon autografts have been associated with higher risks of cyclops syndrome [19, 29]. Since these grafts were used more selectively in primary ACLR in our centre, they were excluded to avoid introducing selection bias.

Age was not identified as a risk factor in this study. The literature remains inconsistent, with some studies reporting that older age (>27 years) may be associated with an increased risk of recurrent cyclops syndrome, but no association was found for primary cyclops lesion, as in this study [19].

Other potential cyclops syndrome risk factors remain debated, such as a narrow intercondylar notch, knee hyperextension, excessively anterior tibial and femoral tunnel placement or concomitant meniscal repair [1, 5, 9, 14, 29, 32, 36]. The only widely accepted risk factor is the presence of an early postoperative extension deficit associated with arthrogenic muscle inhibition [9, 14].

Recurrence of cyclops lesions has also been reported in the literature, but no recurrence was observed in the present study [19]. Identified risk factors for recurrent cyclops syndrome include older age, use of patellar tendon grafts, absence of a LEAP, shorter time interval between ACLR and initial cyclops surgery and residual extension deficit at 6 weeks after arthrolysis [19]. The absence of recurrence in this cohort is likely related to the limited number of cyclops cases, the absence of patellar tendon grafts and the relatively early surgical management, which may have reduced the risk of recurrence.

This study has several limitations. First, its retrospective design introduces inherent biases. Approximately 17% of patients were lost to follow‐up, although this remains acceptable given the minimum 6‐year follow‐up and the young population. Among patients with suspected cyclops syndrome who did not return to our centre, confirmation of diagnosis was not possible. No histological confirmation of the nodules was performed. The relatively low prevalence of cyclops syndrome limited subgroup analyses and reduced statistical power, increasing the risk of type II error. Consequently, these results should be interpreted with caution, and larger prospective cohorts are required to confirm these findings and also to further investigate the influence of meniscectomy on functional tibial slope and the development of cyclops syndrome. Nevertheless, all eligible patients were included, and the surgical procedures were performed by an experienced operator, thereby reducing potential variability in tunnel positioning. Finally, information on early postoperative extension deficits, weight‐bearing protocol, specific meniscal tear patterns and repair techniques, arthrogenic muscle inhibition, notch morphology and tunnel placement was not available, although these may play an important role in cyclops pathogenesis.

## CONCLUSION

Neither increased PTS nor SATT was associated with a higher prevalence of cyclops syndrome following ACLR with hamstring autograft. These preoperative parameters should not alert clinicians to an increased risk of postoperative cyclops syndrome.

## AUTHOR CONTRIBUTIONS

David Mazy, Nicolas Cance and Lucia Angelelli drafted the manuscript. David Mazy, Nicolas Cance and David Henri Dejour were responsible for the research design. David Mazy, Nicolas Cance, Lucia Angelelli, Tomas Pineda and Andrea Pintore were responsible for data acquisition. David Mazy, Nicolas Cance, Lucia Angelelli, Tomas Pineda, Andrea Pintore and David Henri Dejour analysed and interpreted the data. All authors reviewed and approved the final manuscript.

## ETHICS STATEMENT

The study was approved by the institutional ethical board (No. COS‐RGDS‐2020‐03‐006‐DEJOUR‐D).

## CONFLICT OF INTEREST STATEMENT

David Henri Dejour has received royalties from Arthrex, Science & BioMaterials (SBM), and Corin and consulting fees from Smith & Nephew. The remaining authors declare no conflicts of interest.

## Data Availability

The data that support the findings of this study are available on request from the corresponding author. The data are not publicly available due to privacy or ethical restrictions.

## References

[1] Ahn JH , Yoo JC , Yang HS , Kim JH , Wang JH . Second‐look arthroscopic findings of 208 patients after ACL reconstruction. Knee Surg Sports Traumatol Arthrosc. 2007;15(3):242–248. 17028869 10.1007/s00167-006-0177-8

[2] Brazier J , Migaud H , Gougeon F , Cotten A , Fontaine C , Duquennoy A . Evaluation of methods for radiographic measurement of the tibial slope. A study of 83 healthy knees. Rev Chir Orthop Reparatrice Appar Mot. 1996;82(3):195–200. 9005456

[3] Cance N , Dan MJ , Pineda T , Demey G , Dejour DH . Radiographic investigation of differences in static anterior tibial translation with axial load between isolated ACL injury and controls. Am J Sports Med. 2024;52(2):338–343. 38166410 10.1177/03635465231214223

[4] Colombet P , Dejour D , Panisset J‐C , Siebold R . Current concept of partial anterior cruciate ligament ruptures. Orthop Traumatol Surg Res. 2010;96(8 Suppl):S109–S118. 21056025 10.1016/j.otsr.2010.09.003

[5] Cong T , Dadoo S , Inoue J , Nukuto K , Grandberg C , Hughes JD , et al. Risk profile for cyclops syndrome necessitating reoperation after anterior cruciate ligament reconstruction. Am J Sports Med. 2025;53(13):3098–3107. 41105526 10.1177/03635465251376585

[6] Csintalan RP , Inacio MCS , Funahashi TT , Maletis GB . Risk factors of subsequent operations after primary anterior cruciate ligament reconstruction. Am J Sports Med. 2014;42(3):619–625. 24335588 10.1177/0363546513511416

[7] Dan MJ , Cance N , Pineda T , Demey G , Dejour DH . Four to 6° is the target posterior tibial slope after tibial deflection osteotomy according to the knee static anterior tibial translation. Arthroscopy. 2024;40:846–854. 37479151 10.1016/j.arthro.2023.07.007

[8] Dejour D , Zaffagnini S , Ntagiopoulos PG , Grassi A , Muccioli GMM , Marcacci M . ACL reconstruction with extra‐articular plasty. In: Siebold R , Dejour D , Zaffagnini S , editors. Anterior cruciate ligament reconstruction: a practical surgical guide. Berlin, Heidelberg: Springer; 2014. p. 299–316.

[9] Delaloye J‐R , Murar J , Vieira TD , Franck F , Pioger C , Helfer L , et al. Knee extension deficit in the early postoperative period predisposes to cyclops syndrome after anterior cruciate ligament reconstruction: a risk factor analysis in 3633 patients from the SANTI study group database. Am J Sports Med. 2020;48(3):565–572. 31930921 10.1177/0363546519897064

[10] Eckenrode BJ . An algorithmic approach to rehabilitation following arthroscopic surgery for arthrofibrosis of the knee. Physiother Theory Pract. 2018;34(1):66–74. 28862529 10.1080/09593985.2017.1370754

[11] Foissey C , Abid H , Freychet B , Sonnery‐Cottet B , Thaunat M , Fayard J‐M . Postoperative regular use of a self‐rehabilitation mobile application for more than two weeks reduces extension deficit and cyclop syndrome after anterior cruciate ligament reconstruction. J Exp Orthop. 2023;10(1):14. 36757506 10.1186/s40634-023-00578-z PMC9911572

[12] Getgood AMJ , Bryant DM , Litchfield R , Heard M , McCormack RG , Rezansoff A , et al. Lateral extra‐articular tenodesis reduces failure of Hamstring tendon autograft anterior cruciate ligament reconstruction: 2‐year outcomes from the STABILITY study randomized clinical trial. Am J Sports Med. 2020;48(2):285–297. 31940222 10.1177/0363546519896333

[13] Gohil S , Falconer TM , Breidahl W , Annear PO . Serial MRI and clinical assessment of cyclops lesions. Knee Surg Sports Traumatol Arthrosc. 2014;22(5):1090–1096. 23572043 10.1007/s00167-013-2480-5

[14] Guerra‐Pinto F , Thaunat M , Daggett M , Kajetanek C , Marques T , Guimaraes T , et al. Hamstring contracture after ACL reconstruction is associated with an increased risk of cyclops syndrome. Orthop J Sports Med. 2017;5(1):2325967116684121. 28203602 10.1177/2325967116684121 PMC5298440

[15] Hefti F , Müller W , Jakob RP , Stäubli H‐U . Evaluation of knee ligament injuries with the IKDC form. Knee Surg Sports Traumatol Arthrosc. 1993;1(3–4):226–234. 8536037 10.1007/BF01560215

[16] Jackson DW , Schaefer RK . Cyclops syndrome: loss of extension following intra‐articular anterior cruciate ligament reconstruction. Arthroscopy. 1990;6(3):171–178. 2206179 10.1016/0749-8063(90)90072-l

[17] Jameson SS , Dowen D , James P , Serrano‐Pedraza I , Reed MR , Deehan D . Complications following anterior cruciate ligament reconstruction in the English NHS. Knee. 2012;19(1):14–19. 21216599 10.1016/j.knee.2010.11.011

[18] Mazy D , Cance N , Favroul C , Angelelli L , Beckers G , Dan MJ , et al. The impact of posterior tibial slope and static anterior tibial translation on ACL graft rupture rates after Hamstring autograft reconstruction with lateral extra‐articular tenodesis. Am J Sports Med. 2025;53(10):2379–2386. 40589211 10.1177/03635465251350397

[19] Meissburger V , Lefèvre N , Moussa MK , Bohu Y , Gerometta A , Grimaud O , et al. Recurrence of cyclops syndrome after initial arthrolysis: characteristics and risk factors after ACL reconstruction. Orthop J Sports Med. 2025;13(7):23259671251355126. 40687704 10.1177/23259671251355126 PMC12276482

[20] Miller LL , Lind M , Mechlenburg I , Nielsen TG . Seven percent of primary anterior crucial ligament reconstruction patients have arthroscopic resection of cyclops lesions within 2 years: a cohort study of 2556 patients. Knee Surg Sports Traumatol Arthrosc. 2024;32(6):1455–1461. 38629753 10.1002/ksa.12165

[21] Noailles T , Chalopin A , Boissard M , Lopes R , Bouguennec N , Hardy A . Incidence and risk factors for cyclops syndrome after anterior cruciate ligament reconstruction: a systematic literature review. Orthop Traumatol Surg Res. 2019;105(7):1401–1405. 31405748 10.1016/j.otsr.2019.07.007

[22] Nwachukwu BU , McFeely ED , Nasreddine A , Udall JH , Finlayson C , Shearer DW , et al. Arthrofibrosis after anterior cruciate ligament reconstruction in children and adolescents. J Pediatr Orthop. 2011;31(8):811–817. 22101657 10.1097/BPO.0b013e31822e0291

[23] Peez C , Waider C , Deichsel A , Briese T , Palma Kries LK , Herbst E , et al. Proximal tibial anatomical axis and anterior tibial cortex‐based measurements of posterior tibial slope on lateral radiographs differ least from actual posterior tibial slope—a biomechanical study. J Exp Orthop. 2024;11(4):e70108. 39664925 10.1002/jeo2.70108 PMC11632255

[24] Rougereau G , Rollet ME , Pascal‐Moussellard H , Granger B , Khiami F . A tight anterosuperior intercondylar notch may increase the risk of cyclops syndrome after anterior cruciate ligament reconstruction using a quadruple semi‐tendinosus short autograft. Orthop Traumatol Surg Res. 2025;111(2):103918. 38876210 10.1016/j.otsr.2024.103918

[25] Rousseau R , Labruyere C , Kajetanek C , Deschamps O , Makridis KG , Djian P . Complications after anterior cruciate ligament reconstruction and their relation to the type of graft: a prospective study of 958 cases. Am J Sports Med. 2019;47(11):2543–2549. 31403824 10.1177/0363546519867913

[26] Said S , Christainsen SE , Faunoe P , Lund B , Lind M . Outcome of surgical treatment of arthrofibrosis following ligament reconstruction. Knee Surg Sports Traumatol Arthrosc. 2011;19(10):1704–1708. 21432620 10.1007/s00167-011-1472-6

[27] Salmon LJ , Heath E , Akrawi H , Roe JP , Linklater J , Pinczewski LA . 20‐year outcomes of anterior cruciate ligament reconstruction with Hamstring tendon autograft: the catastrophic effect of age and posterior tibial slope. Am J Sports Med. 2018;46(3):531–543. 29244525 10.1177/0363546517741497

[28] Sanders TL , Kremers HM , Bryan AJ , Kremers WK , Stuart MJ , Krych AJ . Procedural intervention for arthrofibrosis after ACL reconstruction: trends over two decades. Knee Surg Sports Traumatol Arthrosc. 2017;25(2):532–537. 26410093 10.1007/s00167-015-3799-x PMC4936949

[29] Sonnery‐Cottet B , Lavoie F , Ogassawara R , Kasmaoui H , Scussiato RG , Kidder JF , et al. Clinical and operative characteristics of cyclops syndrome after double‐bundle anterior cruciate ligament reconstruction. Arthroscopy. 2010;26(11):1483–1488. 20875722 10.1016/j.arthro.2010.02.034

[30] Stijak L , Radonjić V , Nikolić V , Blagojević Z , Aksić M , Filipović B . Correlation between the morphometric parameters of the anterior cruciate ligament and the intercondylar width: gender and age differences. Knee Surg Sports Traumatol Arthrosc. 2009;17(7):812–817. 19421737 10.1007/s00167-009-0807-z

[31] Tagesson S , Öberg B , Kvist J . Static and dynamic tibial translation before, 5 weeks after, and 5 years after anterior cruciate ligament reconstruction. Knee Surg Sports Traumatol Arthrosc. 2015;23(12):3691–3697. 25261221 10.1007/s00167-014-3279-8

[32] Tan SHS , Lau BPH , Khin LW , Lingaraj K . The importance of patient sex in the outcomes of anterior cruciate ligament reconstructions: a systematic review and meta‐analysis. Am J Sports Med. 2016;44(1):242–254. 25802119 10.1177/0363546515573008

[33] Tarchichi J , Daher M , Bouyge B , Graveleau N , Morvan A , Laboudie P , et al. Good satisfaction and functional outcomes after arthroscopic debridement of cyclops syndrome post‐anterior cruciate ligament reconstruction: analysis of 197 patients of the MERIscience cohort. Knee Surg Sports Traumatol Arthrosc. 2025:70188. 10.1002/ksa.70188 41235440

[34] Tollefson LV , Rasmussen MT , Guerin G , LaPrade CM , LaPrade RF . Slope‐reducing proximal tibial osteotomy improves outcomes in anterior cruciate ligament reconstruction patients with elevated posterior tibial slope, especially revisions and posterior tibial slope ≥12. Arthroscopy. 2024;41(8):3184–3195. 39536996 10.1016/j.arthro.2024.10.048

[35] Voskuijl T , Webster KE , Whitehead TS , Klemm HJ , Batty LM , Feller JA . The addition of a lateral extra‐articular procedure to a primary anterior cruciate ligament reconstruction is associated with an increased rate of further surgery for cyclops lesions and restricted range of motion. Knee Surg Sports Traumatol Arthrosc. 2025:70089. 10.1002/ksa.70089 PMC13502448 41081577

[36] Wang J , Ao Y . Analysis of different kinds of cyclops lesions with or without extension loss. Arthroscopy. 2009;25(6):626–631. 19501293 10.1016/j.arthro.2008.12.006

